# Environmental health, economy, and amenities interactively drive migration patterns among China's older people

**DOI:** 10.3389/fpubh.2024.1354071

**Published:** 2024-04-10

**Authors:** Hongjie Wang, Xiaolu Gao, Guili Liu, Fuyuan Wang, Mark W. Rosenberg

**Affiliations:** ^1^College of Applied Arts and Science, Beijing Union University, Beijing, China; ^2^School of Architecture and Urban Planning, Beijing University of Civil Engineering and Architecture, Beijing, China; ^3^Institute of Geographic Sciences and Natural Resource Research, Chinese Academy of Sciences, Beijing, China; ^4^Department of Geography and Planning, Queen's University, Kingston, ON, Canada

**Keywords:** older people, migration pattern, environmental health, interaction, China

## Abstract

The increasing number of older adult migrants is rapidly changing regional demographic and social structures in China. There is an urgent need to understand the spatial patterns and factors that influence older adults to migrate, especially the role of environmental health. However, this issue has been under-studied. This study focused on intra-provincial and inter-provincial older adult migrants as research subjects, estimated their spatial concentration index based on the iterative proportional fitting approach, and explored the factors influencing their migration using the GeoDetector Model. The results showed the following: (1) In 2015, more than 76% of inter-provincial older adult migrants were distributed in Eastern China, and most intra-provincial older adult migrants were scattered in sub-provincial cities. (2) Compared to factors relating to economy and amenities, environmental health by itself played a relatively weak role in the migration of older adults, but the interaction among environmental health, economy, and amenities was a key driving force of older adult migration. (3) There were significant differences in the dominant environmental health factors between inter-provincial migration and intra-provincial migration, which were temperature and altitude, respectively. Our findings can help policymakers focus on the composition of older adult migrants based on urban environmental health characteristics and rationally optimize older adult care facilities to promote supply-demand matching.

## Introduction

On a global scale, older adult migrants exhibit a clustering and disassortative pattern, implying a globalized and multi-polarized trend (1). In the West, people move more frequently and across greater distances, including both transnational and regional migration. In China, there are fewer transnational migrations, but many regional migrations. As the country with the largest number of older people in the world, China's older adult migrants should not be underestimated. Since 1978, with the economic reforms shifting from a planned economy to a market economy and the relaxation of rural-to-urban migration policy controls (2), migration among older adults has also grown rapidly. In 2010, 2015, and 2020, the number of older adult migrants in China was 10.61, 17.78, and 33.27 million, respectively (National Bureau of Statistics data, https://www.stats.gov.cn), tripling in 10 years. The proportion of older adult migrants among total migrants also went up from 4.80% in 2010 to 5.96% in 2015 and 8.85% in 2020. This trend is bound to increase continuously with reductions in travel costs and increased pursuits of individual comfort and health (3–5). Compared with changes in the natural growth rate, older adult migrants have become a more substantial component that impacts aging spatial patterns (6). Information about older adult migrants, in particular, estimates of their number, their origin and destination, and their driving conditions are much needed for the planning of urban services and infrastructure. Understanding migration patterns across spatial scales—including inter-provincial and intra-provincial migration—is thus fundamental for policy design.

Both attractive and unattractive characteristics of cities influence migratory behavior (7). Ideal places encourage moving in and hinder moving out, whereas less desirable places do the opposite (8). Due to inconsistent preferences and composition of the population, research studies on the influencing factors have produced inconsistent and even contradictory results. The regional economy has always played an important role in population migration (9, 10). If the migration benefit is larger than the migration cost, it will promote migration, for instance, a higher annual net immigration rate is related to the GDP and disposable income (11, 12). Similarly, lower living costs are yet another key factor driving migration (13). The size of the city also plays a role in a person's decision to move and choose a destination (14). Some scholars have suggested that large cities have the highest attractivity (15), while others hold the opposite opinion (16). Regardless, the natural conditions of a place are fundamental to its character and attributes. People generally prefer places with plenty of vegetation (17, 18) and a comfortable climate (19–21). On the contrary, environmental pollution encourages people to migrate out (22, 23). Although natural amenities are attractive to populations, numerous scholars believe that people prefer to live in more urbanized and accessible environments with convenient facilities. For instance, public medical service facilities are one of the most basic needs that people seek (24). Furthermore, public transportation that aids in mobility and accessibility (25) and places with numerous parks are more attractive to people (26, 27).

However, the existing research on migration mainly focuses on migrant workers and young migrants, while the literature on the spatial distribution and migration patterns of older adult migrants is insufficient. Older adult migrants have withdrawn from the labor market and do not look for employment opportunities; instead, they have a higher demand for public health resources and nursing systems. Therefore, the redistribution of older adults imposes significant challenges to public healthcare systems. It is possible that public health resources may be without use in the emigration area while also being in short supply in the immigration area. Some cities have made efforts to build and expand their healthcare infrastructure, promoting the healthcare industry as a new engine of economic growth, but large investments have not yielded their expected effects. For example, the bed utilization rate of pension institutions in Beijing was only 39%, according to a market survey in 2020. Many construction project failures have been attributed to spatial dislocation, which is an underestimation or overestimation of older adult migrants on the corresponding spatial scale. In addition, older people are increasingly concerned about environmental health (28), which makes it necessary to study the driving mechanism of environmental health on older adult migration and the interaction between environmental health and other factors. Therefore, in this study, we first analyzed the spatial distribution of older adult migrants using a spatial concentration index based on a simulated dataset of older adult migrants in prefecture-level cities. We then explored the migration mechanism of older adults from environmental health, economy, and amenities factor perspectives using the GeoDetector model.

## Materials and methods

### Data resources

In China, public services are provided based on where the individual resides. We define older adult migrants as residents over 60 years who have lived in the locale for more than 6 months (29). The age is the cutoff used by the National Bureau of Statistics for population statistics for those living without obtaining a local hukou (household registration). Considering that the Chinese government usually formulates specific policies by provinces based on the central policy, we adopted the province as the boundary to study migration, categorizing older adult migrants into two groups based on their migration patterns—inter-provincial migration and intra-provincial migration. We define inter-provincial migration as the migration from other prefecture-level cities within the same province, excluding the migration from the same prefecture-level city.

The China *National Population Census* and the *China Intercensal Population Sample Survey of One-Percent* (National Bureau of Statistics data, https://www.stats.gov.cn) offer the most accurate and credible data for up-to-date population statistics. Although the *National Population Census* data in 2020 has been released, some of the required data are lacking and are affected by the outbreak of COVID-19. Therefore, we still simulated the dataset of older adult migrants in prefecture-level cities using the 2015 *Intercensal Population Sample Survey of One-Percent*. It includes 246 prefecture-level cities in 27 provinces, excluding the four provinces of Anhui, Inner Mongolia, Tibet, and Shandong, which have no data. It is worth noting that the original data must be preprocessed before simulation. First, it should be converted to the resident population according to the sampling ratio, which is the ratio of the sampled population of each province to the resident population of each province. Second, the floating population in the same city needs to be deleted since the population with separated households within municipal jurisdiction still belongs to the local population. Finally, the floating population of children, youth, and older people needs to be separated by age and household registration status.

### Variables

#### Spatial concentration index

After obtaining the estimated numbers of older adult migrants in each city through the iterative proportional fitting (IPF) model, we divided the estimated data into two categories—inter-provincial older adult migrants and intra-provincial older adult migrants. The impact of older adult migrants on local older residents was assumed to be “a,” and the impact of older adult migrants on the local total population was assumed to be “b.” When measuring the comprehensive impact of older adult migrants on the local older residents and the local total population, the geometric mean ($\sqrt{ab}$) is relatively less affected by extreme values and multiples of enlargement. Therefore, we constructed the spatial concentration index (Y) as the geometric mean of the ratio of older adult migrants to the local older residents and the ratio of older adult migrants to the local total population (Equation 1). The spatial concentration index reflects the impact of older adult migrants on the population structure of the destination, quantifying the burden of migrants on local older adult care, and indicates the attractiveness of the destination to older adult migrants. The specific formula is as follows:


(1) $$\begin{array}{l} Y_{i}=\sqrt{{\frac{OM_{i}}{O_{i}}}^{*}\frac{OM_{i}}{P_{i}}}=\sqrt{{\frac{OM_{i}}{P_{i}^{*}A}}^{*}\frac{OM_{i}}{P_{i}}}={\frac{1}{\sqrt{A}}}^{*}\frac{OM_{i}}{P_{i}}, \end{array}$$

where *Y*_*i*_ is the older migration coefficient (that is, spatial concentration index), *OM*_*i*_ is the number of older adult migrants, *O*_*i*_ is the local older residents, *P*_*i*_ is the total population, and A is the aging rate, all in i area. A city with a large *Y*_*i*_ is a popular city for older adult migrants, and correspondingly, older adult migrants increase their pressure on pensions.

#### Driving factors

The environment is closely related to human health, and an appropriate environment is beneficial to the physical and mental health of the people residing there. In particular, older adults are a relatively vulnerable group with declining physical functions. The impact of environmental factors on older adults may be more obvious and serious during migration. Environmental health factors are defined as environmental factors affecting population health (30). In this study, air quality, elevation, humidity, and temperature (31) were selected as proxy variables of environmental health factors (Table 1).

**Table 1:** Indicators of the key factors influencing migration among older adult migrants.

**Factors**	**Variables**	**Definitions and description**	**Data resources**
Environmental health	Air quality	Average annual PM_2.5_ (ug/m^3^)	http://www.cnemc.cn/
	Elevation	Elevation of the city (m)	http://www.gscloud.cn
	Humidity	Average relative humidity (%)	https://www.resdc.cn/
	Temperature	Winter average temperature (°C)	https://www.resdc.cn/
Economy	PGDP	Per capita GDP (yuan)	China Statistical Yearbook 2015
	Income	Per capita disposable income (yuan)	
	Consumption	Consumer goods retail sales (100 million yuan)	
	Expenditure	Public budget expenditure (100 million yuan)	
Amenities	Public health	The number of doctors per 1,000 people	China Statistical Yearbook 2015
	Public transport	Public transport vehicles per 10,000 people	China Statistical Yearbook 2015
	Housing price	Housing prices in each city (yuan/m^2^)	http://www.sic.gov.cn/
	Green	The ratio of green space to built-up area	China Statistical Yearbook 2015

Continuous variables should be classified using the natural breaks method before performing the GeoDetector analysis.

In addition, factors relating to economy and amenities were selected as control variables. Factors relating to economy, including per capita GDP, income level, consumption level, and public expenditure, have generally been used in previous studies to reflect the level of socioeconomic development. Factors relating to amenities include public healthcare, public transportation, housing prices, and urban green spaces. While healthcare is an indispensable need for older adult migrants, transportation and housing are considered fundamental needs, and urban green spaces provide a place for leisure and entertainment.

### Methods

#### The iterative proportional fitting model

The iterative proportional fitting model is a mathematical scaling procedure that estimates cell probabilities in a contingency table based on observations that are subject to constraints, including known and fixed marginal row and column totals. It was first proposed by Deming and Stephan (32). IPF is employed in various disciplines but has been particularly useful in census-related analysis. A two-dimensional table of data is adjusted so that its row and column totals agree with constraining row and column totals obtained from alternative sources (33). IPF acts as a weighting system whereby the original table values are gradually adjusted through repeated calculations to fit the row and column constraints. The resultant table of data is a “joint probability distribution” of “maximum likelihood estimates” obtained when the probabilities converge within an acceptable (pre-defined) limit (34). In the two-dimensional table in our study, the rows designate the “total migrants in each prefecture-level city” and the columns indicate “migrants in each province divided by age,” as obtained from the 2015 *Intercensal Population Sample Survey of One-Percent*. The seeds are the total number of older adult residents of each prefecture-level city unit in 2015. These data were obtained from the *China Statistical Yearbook 2015*, published for each province and autonomous region.


(2) $$\begin{array}{l} P_{ij}\left(k+1\right)=\frac{P_{ij}\left(k\right)}{\underset{i}{\sum }P_{ij}\left(k\right)}Q_{i}, \end{array}$$

(3) $$\begin{array}{l} P_{ij}\left(k+2\right)=\frac{P_{ij}\left(k+1\right)}{\underset{i}{\sum }P_{ij}\left(k+1\right)}Q_{j}, \end{array}$$

where *P*_*ij*_(*k*) is the matrix element in row i, column j, and iteration k. *Q*_*i*_ and *Q*_*j*_ are the pre-defined row totals and column totals, respectively, delineating the migrants by age and by city, respectively.


(4) $$\begin{array}{l} \underset{j}{\sum }P_{ij}\left(m\right)=Q_{i}\;and\;\underset{i}{\sum }P_{ij}\left(m\right)=Q_{j}, \end{array}$$

where Equations (2, 3) are employed iteratively to estimate new cell values and will theoretically stop at iteration m (Equation 4). At this point, the final estimated number of older adult migrants in each prefecture-level city has been obtained.

#### The GeoDetector model

GeoDetector has been widely used as a statistical method to explore spatial heterogeneity and reveal the driving forces (35). The basic premise is that, if an independent variable (X) has a significantly similar spatial distribution to the dependent variable (Y), the coupling relationship between X and Y does exist. There are several advantages of the GeoDetector method: the relationship between X and Y does not require linear assumptions, collinearities of multiple variables will not influence the results, and the interaction between two independent variables on Y can be measured (36). Since the independent variables used in GeoDetector must be categorical variables, the natural breaks classification method should be applied to convert the drivers' data. GeoDetector includes four modules: factor, interaction, ecological, and risk detector, which can be obtained at http://www.geodetector.org/. The factor detector uses the q-value to quantify the impact of the factors, which is given as follows:


(5) $$\begin{array}{l} q=1-\frac{\overset{L}{\underset{h=1}{\sum }}N_{h}\sigma_{h}^{2}}{N\sigma^{2}}=1-\frac{SSW}{SST}, \end{array}$$

(6) $$SSW=\sum_{h=1}^{L}N_{h}\sigma_{h}^{2}=\sum_{h=1}^{L}\sum_{i}^{N_{h}}{\left(Y_{hi}-Y_{h}\right)}^{2},$$

(7) $$SST=\;N\sigma^{2}=\sum_{i}^{N}{\left(Y_{i}-Y\right)}^{2},$$

where q is the power of determinant (Equations 5–7); h = 1, …, L is the layer of the independent variable X; Nh and N are the numbers of prefecture-level cities in the layer h and the total region, respectively; $\sigma_{h}^{2}$ and σ^2^ are the variance of the dependent variable Y in the h layer and the total region, respectively; SSW is within the sum of squares, and SST is the total sum of squares; *Y*_*hi*_ and *Y*_*i*_ denote the dependent variable value of unit i in the h layer and the total region;*Y*_*h*_ and *Y* denote the mean value of the dependent variable in the h layer and the total region. The value range of q is [0,1], in which a larger *q* value indicates that the factor explains more of the Y.

The interaction detector quantifies the interaction between two factors to assess whether the factors X1 and X2 weaken or enhance each other or whether they are independent (Table 2). The *q*-values of two factors X1 and X2 are obtained first, and then, their interaction *q*-value (X1∩X2) is calculated. The q(X1∩X2) is the *q*-value of the new polygon formed by the tangent of the X1 and X2 layers. As shown in Table 2, the relationship between the two factors can be divided into five categories by comparing q(X1), q(X2), and q(X1∩X2).

**Table 2:** Categories of interaction relationship between two factors.

**Interaction categories**	**Description**
Non-linear weaken	q(*X*_1_∩*X*_2_) < min (q(*X*_1_), q(*X*_2_))
Uni weaken	Min (q(*X*_1_), q(*X*_2_)) < *q*(*X*_1_∩*X*_2_) < max (q(*X*_1_), q(*X*_2_))
Bi enhance	Max (q(*X*_1_), q(*X*_2_)) < *q*(*X*_1_∩*X*_2_) < q(*X*_1_)+ q(*X*_2_)
Independent	q(*X*_1_∩*X*_2_) = q(*X*_1_)+ q(*X*_2_)
Non-linear enhance	q(*X*_1_∩*X*_2_) >q(*X*_1_)+ q(*X*_2_)

## Results

### Descriptive statistics

Although the number of older adult Chinese migrants was 17.78 million in 2015 according to the National Bureau of Statistics, we finally obtained 10.45 million older adult migrants from 246 prefecture-level cities through the IPF model simulation, which was due to the lack of specific data in some provinces. Among them, 2.34 million were inter-provincial older adult migrants, and 8.11 million were intra-provincial older adult migrants.

**Figure 1:**
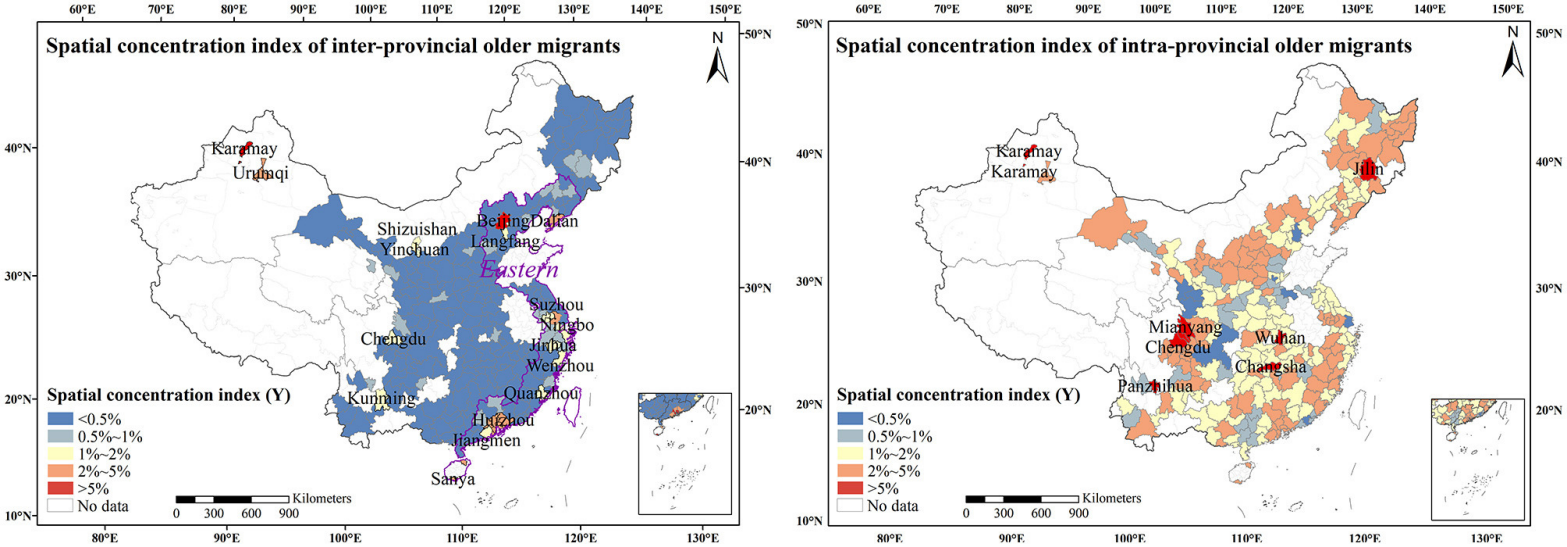
Spatial distribution of inter-provincial and intra-provincial older adult migrants based on the spatial concentration index in 2015.

The top 10 prefecture-level cities with the highest numbers of inter-provincial older adult migrants include Shanghai, Beijing, Tianjin, Guangzhou, Suzhou, Foshan, Shenzhen, Dongguan, Chengdu, and Dalian (Figure 1). The spatial concentration index of inter-provincial older adult migrants is divided into five types: < 0.5, 0.5–1, 1–2, 2–5, and >5%. The cities with spatial concentration index >5% are Beijing (6.57%) and Karamay (6.41%). The cities with spatial concentration index of 2–5% are mainly distributed in the Pearl River Delta (Dongguan, Shenzhen, Zhongshan, Foshan, Zhuhai, Guangzhou, and Huizhou), Hainan Island (Haikou and Sanya), Suzhou, Urumqi, and Dalian. The cities with a spatial concentration index of 1–2% include the Yangtze River Delta (Wuxi, Jiaxing, Ningbo, Jinhua, Quanzhou, Wenzhou, and Changzhou), Jiangmen, Shizuishan, Yinchuan, Kunming, Chengdu, Xiamen, and Langfang. There are 26 cities with a spatial concentration index of 0.5–1%. The cities with a spatial concentration index of < 0.5% accounted for 78.05%. In terms of spatial distribution, the eastern region attracted most of the inter-provincial older adult migrants. Except for traditionally popular municipalities and provincial capitals, we can see that the Yangtze River Delta, Pearl River Delta, and Hainan Island have gradually become hot destinations in the inter-provincial migration for older people, exhibiting clustering and multipolarity characteristics (37).

The top 10 prefecture-level cities with the highest numbers of intra-provincial older adult migrants include Chengdu, Chongqing, Wuhan, Guangzhou, Changsha, Changchun, Mianyang, Shanghai, Xi'an, and Zhengzhou. The spatial concentration index of intra-provincial older adult migrants is divided into five types: < 0.5, 0.5–1, 1–2, 2–5, and >5%. The cities with the spatial concentration index of >5% are Chengdu (9.61%), Panzhihua (6.52%), Wuhan (6.03%), Mianyang (5.98%), Changsha (5.61%), Karamay (5.27%), Jilin (5.22%), and Deyang (5.15%). Among them, Chengdu, Wuhan, and Changsha are provincial capitals; Panzhihua is a famous steel city; Mianyang is an important production base for the electronic industry; and Karamay, Jilin, and Deyang are important industrial cities in China. There are 106 prefecture-level cities with a spatial concentration index of 2–5%, 94 prefecture-level cities with a spatial concentration index of 1–2%, 29 prefecture-level cities with a spatial concentration index of 0.5–1%, and nine prefecture-level cities with a spatial concentration index of < 0.5%. From the perspective of spatial distribution, older adults migrating into the province tend to move to cities with relatively good development in their province, such as sub-provincial cities and cities with industrial support, consistent with previous studies (38).

### Influencing factors

The inter-provincial attractive factors are shown in Table 3 based on the *q*-value of their spatial heterogeneity (*p* < 0.01). For the environmental health factors, altitude and temperature explained 10.2 and 9% of the older adults' inter-provincial migration, while air quality and humidity were not significant. It indicated that altitude and temperature alone played a significant but relatively weak driving role in the older adults' inter-provincial migration. For the economy factors, the *q*-value of per capita GDP was 31.9%, and the *q*-values of income, expenditure, and consumption were all above 20%, suggesting that the economic development level of the destination is an important factor for older adults who plan to migrate across provinces. With regard to amenities, housing price was the highest explanatory factor, with a *q*-value of 34.5%. In China, cities with higher housing prices usually mean that they have relatively complete amenities or tourist attractions. The *q*-values of public transport and public health were 28.9 and 19.3%, respectively, indicating that ample public transportation and healthcare play an important role in inter-provincial migration. Meanwhile, the *q*-value of 7% showed that urban green space alone was not a very important factor in the inter-provincial destination choice for older adults.

**Table 3:** Determinant power (*q*) of influencing factors on inter- and intra-provincial destinations.

**Factors**	**Variables**	**Inter-provincial migration**		**Direction**	**Intra-provincial migration**		**Direction**
		***q***	***p***		***q***	***p***	
Environmental health	Air	0.048	0.051	/	0.061	0.015	+
	Elevation	0.102	0.000	Non-linear	0.121	0.000	Non-linear
	Humidity	0.018	0.509	/	0.015	0.656	/
	Temperature	0.090	0.000	Non-linear	0.094	0.000	Non-linear
Economy	PGDP	0.319	0.000	+	0.123	0.000	+
	Income	0.280	0.000	+	0.129	0.000	+
	Consumption	0.200	0.000	+	0.043	0.478	/
	Expenditure	0.209	0.000	+	0.095	0.052	/
Amenities	Public health	0.193	0.000	+	0.215	0.000	+
	Public transport	0.289	0.000	+	0.112	0.012	/
	Housing price	0.345	0.000	+	0.071	0.092	/
	Green	0.071	0.010	/	0.027	0.367	/

There is heterogeneity in the attractiveness factors for inter-provincial and intra-provincial migration, as shown in Table 3. For the environmental health factors, altitude and temperature alone played a significant but relatively weak driving role in intra-migration, with *q*-values of 12.1 and 9.4%, respectively. Air quality and humidity were not significant factors. For the economy factors, per capita disposable income and per capita GDP were the two main economic factors of intra-provincial migration, with *q*-values of 12.9 and 12.3%, respectively. When older adults migrated within the same province, they were more concerned about the destination's economic and income levels but not the consumption and expenditure levels. Regarding available amenities, the condition of public healthcare played an important role in intra-migration, with an explanation power of 21.5%. Other amenities factors did not play a significant role in the intra-provincial older migration.

This study showed that temperature and altitude were the main environmental health factors with a non-linear correlation for inter- and intra-provincial migration of older adults, while humidity and air quality did not play a significant role. However, the explanation power of temperature or altitude was ~10%, which was far lower than that of economy and amenities factors. Economy factors, especially per capita GDP and per capita disposable income, will influence the moving of older adults to a certain extent, irrespective of whether it is inter-provincial or intra-provincial migration. Interestingly, there are significant differences in amenities factors. Among them, housing prices and public transport play a role in promoting the inter-provincial migration of older adults but not in the intra-provincial migration. On the other hand, public health plays a role in promoting intra-provincial migration but has a relatively lower impact on inter-provincial migration.

### Interaction among environmental health, economy, and amenities factors

**Figure 2:**
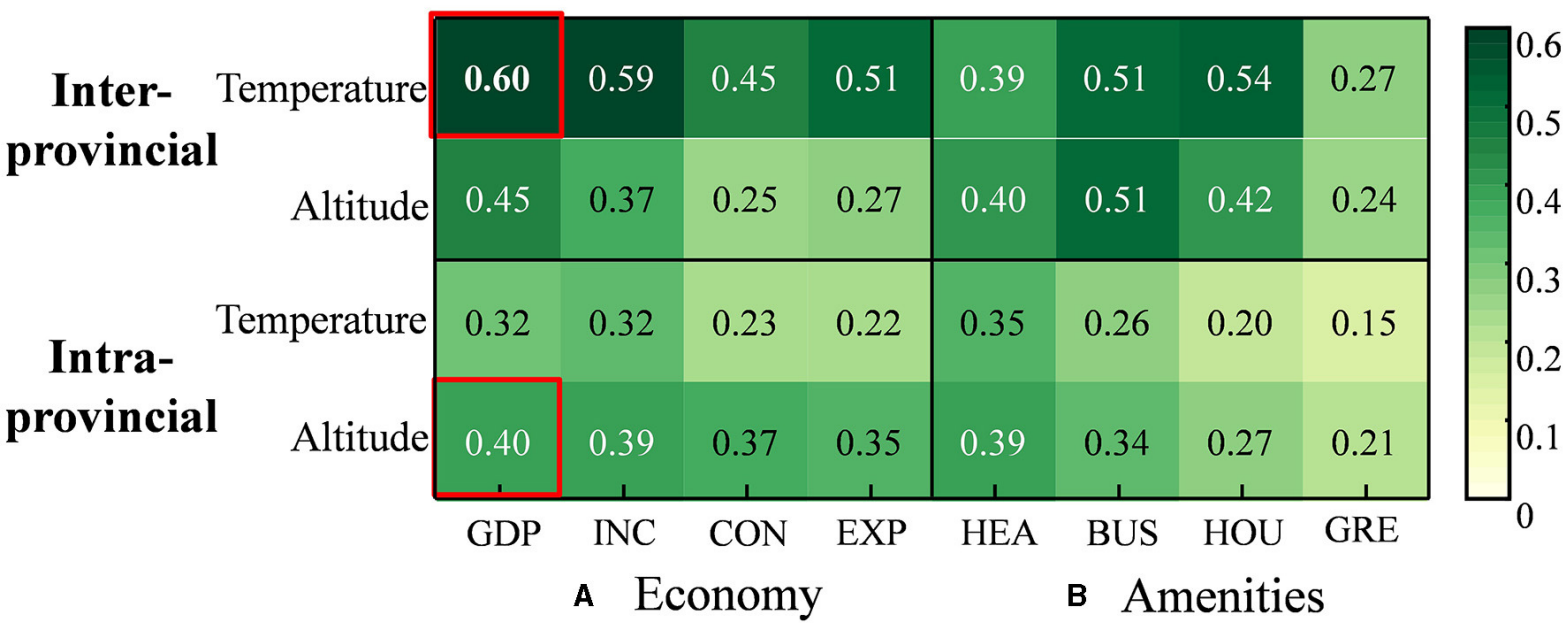
Interaction among environmental health, economy, and amenities factors. **(A)** Economy. **(B)** Amenities.

The effect of interaction among environmental health, economy, and amenities factors on the spatial concentration index was calculated using the interaction detector. There is a synergistic interaction mechanism with factors for the spatial concentration index of both inter-provincial and intra-provincial older adult migrants. The interaction detector showed that the *q*-value of each pair of factors was greater than that of a single factor. The main factors that influenced the spatial concentration index of older adult migrants mainly manifested as a combination of environmental health–economy–amenities interactions (Figure 2).

In the inter-provincial migration pattern, the q(temperature ∩ any economy or amenities factor) was greater than the sum of q(temperature) and q(any economy or amenities factor), indicating that the interaction among temperature, economy, and amenities factors was non-linearly strengthened. The q(altitude ∩ any economic or amenities factor) was less than the sum of q(altitude) and q(any economic or amenities factor), showing that their interaction relationship is binary strengthened. Compared to altitude, the interaction between temperature and other factors was more prominent. It is evident that the explanatory power of temperature was < 10% when considered alone, while it significantly increased when interacting with the economy and amenities factors. The q(temperature ∩ PGDP) was 60%, which was much higher than the sum of q(temperature) and q(PGDP) individually, which were 9 and 31.9%, respectively. The q(temperature ∩ housing price) was 54%, higher than the sum of q(temperature) and q(housing price) individually. The interaction among temperature and income, consumption, and public transport all exceeded 50%. It can be said that the interaction among temperature, economy, and amenities factors played a key role in inter-provincial migration.

In the intra-provincial migration pattern, the interaction among environmental health, including altitude and temperature, economy, and amenities factors exhibited non-linear strength. Compared to temperature, the interaction between altitude and other factors was more prominent. The explanatory power of altitude was < 10% when considered alone, while it significantly increased when interacting with economy and amenities factors. Within the same province, the q(altitude ∩ PGDP) was 40%, which was higher than the sum of q(altitude) and q(PGDP) individually, which were 12.1 and 12.3%, respectively. The q(altitude ∩ public health) was 39%, higher than the sum of q(altitude) and q(public health) individually. It can be preliminarily concluded that the interaction among altitude, economy, and amenities factors contributed significantly to intra-provincial migration.

## Discussion and implications

### Discussion

The spatial patterns of migration are concentrated manifestations of older adult migrants' behaviors. In 2015, there were 10.45 million older adult migrants in the 246 prefecture-level cities in China, including 2.34 million inter-provincial older adult migrants and 8.11 million intra-provincial older adult migrants. Among them, more than 76% of inter-provincial older adult migrants were distributed in Eastern China, mainly in Beijing, Dalian, the Pearl River Delta, the Yangtze River Delta, Hainan Island, and Fujian Province. Intra-provincial older adult migrants were scattered in cities in Central and Western China, such as sub-provincial cities, industrial cities, and tourist cities, which had developed economies and a lack of labor. Yu (15) and Jing (38) confirmed that the intra-provincial migration of older adults was dominant, and the inter-provincial migration showed spatial agglomeration characteristics using different data, which is similar to our findings. Compared with the spatial pattern of all age groups of migrants (37), we found that the destination of inter-provincial older adult migration was tending toward multipolarity.

Temperature, economy, and amenities that are available in the destination interactively drive inter-provincial migration of older adults. Economic factors play an important role in inter-provincial migration, which has been proven by many scholars (11, 12). According to Hedonic pricing (39), housing price is a proxy indicator of the quality of the urban living environment. Cities with high housing prices are usually equipped with excellent urban living conditions (40), which promotes the migration decision-making of the older adult. Considering their poor mobility, public transportation is an important means for the older adult to improve spatial accessibility and access more public service resources. The older adult have a higher demand intensity and higher share of public transportation compared to the non-older adult population (25). Cold spells can increase the risk of cardiovascular and cerebrovascular diseases and fractures (41), and the older adult prefer “escaping extreme low temperatures” (42). The temperature impact alone was relatively weak, while its explanatory power for inter-provincial older adult migrants significantly increases when it interacts with factors relating to economy and amenities. The comprehensive attractiveness of an agreeable temperature (20, 21) and well-developed economy and amenities (19) surpass the geographical constraints of provincial boundaries, and inter-provincial migration has become an important strategy for the older adults.

Altitude, economy, and amenities in destination interactively drive intra-provincial migration among older adults. Intra-provincial older adult migrants pay more attention to the destination's available income. Destinations with more doctors are quite attractive to older adult migrants within the province, considering that public health is directly related to their individual health requirements (24, 43). The availability of private cars and familiarity with geographical locations have reduced the dependence of intra-provincial older adult migrants on public transport when they move within the same province. The impact of altitude alone was relatively weak, but its explanatory power for intra-provincial older adult migrants significantly increased when it interacted with factors relating to the economy and amenities. It indirectly reflects the urbanization process of population transfer from mountainous-hilly areas to plain and coastal areas. Cities with both suitable altitudes and well-developed economies and amenities are more attractive to intra-provincial older adult migrants.

### Policy implications

The spatial pattern of inter-provincial and intra-provincial migration is similar to previous conclusions due to the path dependency effect, where new migrants tend to move along the same migratory paths as their predecessors. Inter-provincial migration of older adults was mainly driven by the interaction among temperature, economy, and amenities. Intra-provincial migration of older adults was mainly driven by the interaction among altitude, economy, and amenities. In the context of aging, it is necessary to reasonably improve public services for older adult migrants in major destinations. Establishing active communication and cooperation between origin and destination governments can avoid spatial mismatch and wastage of resources.

Environmental health, economy, and amenities interactively drive the migration of older adult migrants, in which the relative importance of influencing factors differed between inter-provincial and intra-provincial migration. These findings may provide policymakers with insights into how to attract certain types of older adult migrants in future planning. The patterns and behaviors of older adult migrants should be considered when designing layouts of older adult care facilities. Inter-provincial migrants usually have higher income levels along with higher requirements for amenities. Most intra-provincial migrants are attracted by earnings and adequate healthcare availability. In addition, we suggest that motivations behind migration should be incorporated into optimizing older adult care facilities and layouts to promote supply-demand matches. Convenient public transportation should be considered for “intergenerational care” dominated destinations. Healthcare industries can be developed for “to enjoy the later years” dominated destinations, and foundational services should be guaranteed in “work and business” dominated destinations.

### Limitations and future directions

This study has some limitations that may be addressed in future studies. The first limitation is that the research object (older adult migrants) needs to be further deepened. On the one hand, data on older adult migrants are cross-sectional, and we hope to conduct continuous tracking to analyze their temporal trends. On the other hand, the data on older adult migrants include quantitative and spatial attributes, but they lack details of individual attributes and settlement intentions. The second limitation is the selection of driving factors. This study only selected factors most concerned by the academic community, while the indicators should be further expanded and refined, such as social networks, family ties, and policy interventions. The complex interactions or potential moderating factors among drivers need further exploration. Finally, China is in a period of social transformation, with a unique household registration system and deep family ties. Comparisons between China and Western countries will help to deepen the understanding of aging and social inclusion.

## Conclusion

The study simulated the dataset of older adult migrants at the prefecture-level city scale using the IPF model based on the 2015 *China Intercensal Population Sample Survey of One-Percent*. It categorized older adult migrants into two prevalent patterns—“inter-provincial migration” and “intra-provincial migration”—by taking provinces as the boundary. This study examined the spatial patterns of inter-provincial and intra-provincial migration of older Chinese adults and focused on the interactive impact of environmental health, economy, and amenities on older adult migration through spatial visualization methods and the GeoDetector model. The conclusions are as follows.

First, intra-provincial older adult migrants were more in number and distributed relatively evenly across the sub-provincial cities in Central and Western China. The inter-provincial older adult migrants, on the other hand, were mainly distributed in Eastern China, accompanied by obvious spatial multipolarity. Second, compared to factors relating to the economy and amenities, environmental health was not the main driving factor for older migration from the perspective of a single factor. Even older people who have withdrawn from the mainstream employment market tend to migrate to cities with developed economies and well-equipped amenities. Third, an interesting phenomenon was noticed when the various influencing factors interact. For instance, temperature, economy, and amenities factors in destination interactively drive inter-provincial older adult migration, while altitude, economy, and amenities factors in destination interactively drive intra-provincial older adult migration. The interaction among environmental health, economy, and amenities is the key driving force behind the migration of older adults. There are significant differences in the dominant environmental health factors between inter-provincial and intra-provincial migration.

Overall, the research has certain theoretical and practical significance. First, our findings have strengthened the understanding of environmental health's effect as a migration driver. Having gone through the migration stage of earning income and family reunification, there has been a diversification trend among older adults, such as the urgent need for environmental health and comfort. Our conclusions provide some insights into how environmental health factors affect the migration of older adults and whether there is spatial heterogeneity. Second, the research helps us to understand China's urbanization progress in the middle and later stages of life and provides guidance for creating inclusive, fair, and efficient urban spaces. It is necessary to pay attention to vulnerable migrants for a forward-looking urban layout, avoid wasting public service resources, and improve the effective supply in the origin and destination areas.

## Data availability statement

Publicly available datasets were analyzed in this study. This data can be found at: https://microdata.stats.gov.cn/.

## Author contributions

HW: Investigation, Methodology, Writing – original draft, Data curation, Software, Writing – review & editing. XG: Project administration, Resources, Supervision, Writing – original draft, Methodology. GL: Writing – original draft, Formal analysis, Funding acquisition, Validation, Writing – review & editing. FW: Writing – original draft, Data curation, Software, Visualization. MR: Conceptualization, Formal analysis, Writing – review & editing.
